# Resveratrol Food Supplement Products and the Challenges of Accurate Label Information to Ensure Food Safety for Consumers

**DOI:** 10.3390/nu15020474

**Published:** 2023-01-16

**Authors:** Maja Bensa, Irena Vovk, Vesna Glavnik

**Affiliations:** 1 Laboratory for Food Chemistry, National Institute of Chemistry, Hajdrihova 19, SI-1000 Ljubljana, Slovenia; 2 Faculty of Agriculture and Life Sciences, University of Maribor, Pivola 10, SI-2311 Hoče, Slovenia; 3 Faculty of Health Sciences, University of Ljubljana, Zdravstvena pot 5, SI-1000 Ljubljana, Slovenia

**Keywords:** *trans*-resveratrol, dietary supplements, food safety, regulation, labels, health claims, nutrition claims, novel foods, high-performance thin-layer chromatography, HPTLC

## Abstract

The food supplement market is growing as many consumers wish to complement their nutrient intake. Despite all the regulations in place to ensure food supplements safety, there are still many cases of irregularities reported especially connected to internet sales. Twenty resveratrol food supplement products sold on the Slovenian market were evaluated on their compliance of declared vs. determined resveratrol content, as well as the compliance of labels with the European Union (EU) and Slovenian regulatory requirements. Both the ingredient contents and food information are important parts of food safety. Analyses of 20 food supplements performed using high-performance thin-layer chromatography (HPTLC) coupled with densitometry showed that 95% of products had contents different from what was declared and 55% of products contained higher contents than declared. In 25% of the products the determined content per unit exceeded the maximum level (150 mg/day) specified in EU novel food conditions for food supplement with *trans*-resveratrol. Evaluation of the 20 food supplement labels included mandatory and voluntary food information, food supplement information, novel food information, health claims and nutrition claims. Most labels contained the necessary information, but multiple errors were observed ranging from typos to misleading practices. From a food safety perspective there is still a lot of improvement needed in the field of food supplements.

## 1. Introduction

### 1.1. Food Supplements: Safety and Labels

Food supplements are concentrated sources of nutrients (e.g., vitamins, minerals and other bioactive compounds) taken in dose forms to supplement the normal diet [1]. A healthy, varied diet and a healthy lifestyle are important to maintain good health, but consumers also look at food supplements as additional support for maintaining good health. The market of food supplements is growing on a global scale. The safety of food supplements is of great importance for the health and well-being of consumers. Food supplements are considered as food, and are also regulated as food, even though some forms of food supplements (e.g., capsules, tablets, etc.) can visually resemble medication.

Despite the many regulations in place, there are still many cases of irregularities and food fraud when it comes to food supplements. The 2021 Annual Report of the Alert and Cooperation Network [2] includes information about food noncompliances with EU legislation. The Alert and Cooperation Network (ACN) consists of three networks: the Rapid Alert System for Food and Feed network (RASFF), the Administrative Assistance and Cooperation network (AAC) and the Agri-Food Fraud Network (FFN). The ACN report states that the second highest number (approximately 10%) of noncompliance reports made for food categories in 2021 was for the category of food supplements, dietetic foods and fortified foods [2]. Only the fruit and vegetables category had more reports—about 14% [2]. With regard to food supplements the report also mentions the problematic use of nonauthorized health claims [2]. With regard to food fraud the most concerning practices are: (1) the differences between the declared or marketed contents of substances (e.g., vitamin D) and the actual content in food supplements, (2) the presence of nonauthorized substances that can be harmful to health as well as (3) incomplete or missing information concerning the list of ingredients or responsible food business operators [2]. An increase in the use of food supplements, especially due to internet sales, occurred during the COVID-19 pandemic. Food supplements purchased online often pose a health risk due to many illegal products and food fraud. Half of the RASFF notifications regarding products bought via the internet concerned the food supplement, dietetic foods and fortified foods [2]. While searching for fraudulent practices will probably remain a big challenge for the food sector and regulatory authorities, a lot has already been done in the European Union (EU) to unify the requirements and standards for food products.

EU regulation of food supplements covers several important aspects: food supplements [1], food labeling [3], novel foods [4], as well as health and nutrition claims [5]. Labels are intended to provide the consumer with correct and clear information to help the consumer make informed purchase choices. Labels are not allowed to be misleading or confusing for the consumer. Labels of food supplements include: mandatory information, voluntary information, health and nutrition claims, as well as information required for food supplements and novel foods (if applicable).

### 1.2. Mandatory and Voluntary Food Information

Regulation (EU) No 1169/2011 lists the requirements for food information, both mandatory and voluntary. Food information should be accurate and understandable for consumers. Food labels should not be misleading and should not connect the food’s properties with preventing or treating disease. Regulation (EU) No 1169/2011 lists 12 categories of mandatory information for food products: (1) name of the food, (2) list of ingredients, (3) allergens, (4) quantities of certain ingredients or categories of ingredients, (5) net quantity of the food, (6) date of minimum durability, (7) any special storage conditions and/or conditions of use, (8) name and address of the food business operator, (9) country of origin or place of provenance, (10) instructions for use, (11) nutrition declaration, as well as (12) alcoholic strength by volume for alcoholic beverages [3]. For each of the 12 items there are additional rules, details and conditions for providing the information. The degree to which each of the 12 items is mandatory depends on the food product.

The list of ingredients must include the word “ingredients” in the title. Allergenic ingredients need to be included in the list and written in a different font (e.g., bold). Any nano materials also need to be labeled. Certain information, such as country of origin, is mandatory if without this information the consumer could be misled. Instructions for use are mandatory if the product cannot be used without them. Special storage conditions are also required depending on the product. The nutrition declaration is not used for food supplements [1] except when health or nutrition claims are included on the label [5]. Regulation (EU) No 1169/2011 [3] specifies the daily reference intakes for vitamins and minerals as well as reference intakes for energy and other selected nutrients. Voluntary food label information is also regulated [3] and can include information about the following: unintentional presence of allergens, suitability for vegetarians or vegans, reference intakes for specific population groups as well as absence or reduced presence of gluten. Regulation (EU) No 1169/2011 [3] is clear that voluntary information must be based on scientific evidence, understandable to the consumers and must not overtake the space for mandatory food information. Quite common are claims connected to the absence or reduced presence of gluten or lactose, which is also regulated in other regulations (e.g., Regulation (EU) No 828/2014 [6]).

### 1.3. Health and Nutrition Claims

Food supplement and other food product labels often include health and nutrition claims. The rules about nutrition and health claims are provided in the Regulation (EC) No 1924/2006 [5]. Claims on labels must be comprehensive and scientifically proven. Misleading claims are not allowed. Only authorized claims can be used. Authorized and nonauthorized health claims are available in the EU register of Nutrition and Health Claims [7], which also includes authorized nutrition claims.

Nutrition claims describe properties related to the energy and nutrients or other substances (1) provided, (2) provided in an increased or decreased amount or (3) not provided).

Health claims describe the connection between food and health. Regulation (EC) 1924/2006 also sets the conditions for using nutrition and health claims [5]. The substance for which the claim is made needs to be in a form that can be used by the body and must be present in a sufficient quantity for producing the claimed nutritional or physiological effect. The labels can include health and nutrition claims if the effects they describe are understood by the average consumer [5]. For health claims to be permitted, the labeling needs to include the following four statements: (1) warning for products that are likely to present health risks if consumed to excess, (2) persons who should avoid using the food, (3) the quantity of the food and pattern of consumption required to obtain the claimed beneficial effects, and (4) the importance of a varied and balanced diet, and a healthy lifestyle.

### 1.4. Food Supplements and Novel Foods

To protect consumers from possible misinformation and health risks only food supplements fulfilling the requirements of the Directive 2002/46/EC [1] can be sold in the EU market. The Directive 2002/46/EC [1] lists the vitamins and minerals that can be included among food supplement ingredients together with the permitted sources of vitamins and minerals. The Directive [1] also prescribes units of vitamins and minerals that are to be used on food supplement labels. All food supplements need to be declared as “food supplement” on the label [1]. Advertising of food supplement products is not allowed to claim disease prevention or healing properties. Food supplement labels need to include: (1) the names of the categories of nutrients; (2) the portion of product recommended for daily consumption; (3) a warning not to exceed the stated recommended daily dose; (4) a warning that food supplements should not be used as a substitute for a varied diet; (5) a warning to store the products out of the reach of young children; (6) the amount of minerals or vitamins in specified units and as a percentage of the reference values; and (7) the amount of nutrients or substances with nutritional or physiological effect present in the product and per daily dose.

Sometimes, due to the nature of the food supplement products or their ingredients food supplements are regulated as novel foods. Examples of novel food are resveratrol food supplements with resveratrol from Japanese knotweed extract or microbial source. Novel foods are foods that were not used as human food on a larger scale in the EU before 1997 [8]. The novel foods listed in the Commission Implementing Regulation (EU) 2017/2470 can be sold in the EU. The authorized novel food is *trans*-resveratrol in the food category of food supplements with maximum levels of 150 mg/day. Labels of resveratrol food supplements must include “*trans*-resveratrol” and a statement for patients using medicines to only consume the product under medical supervision.

Despite the regulations in place, many reports of food fraud and misleading practices concerning food supplements have been reported through Rapid Alert System for Food and Feed RASFF [2]. The reports reveal products that were not compliant and harmful to health as well as a lack of control of internet sales. These problems underscore the importance of developing analytical methods that enable fast and effective analysis of food supplements, ensuring consumer’s safety.

Different phenolic compounds are frequently found among the ingredients of food supplements. Resveratrol is a good example as it is included in many food supplement products. Resveratrol is a stilbene with two isomeric forms (*cis* and *trans*). It is also present in different foods (such as grapes, peanuts, pistachio, strawberries, currants and blackberries). Resveratrol has been linked to protection of the heart and blood vessels as well as the following bioactivities: anticarcinogenic; antioxidative; anti-inflammatory; antitumor and antiviral activity [9]. Studies also showed the inhibitory activity of resveratrol against influenza virus replication and the potential of resveratrol in combination with pterostilbene as antiviral compounds to inhibit severe acute respiratory syndrome coronavirus 2 (SARS-CoV-2) infection [10]. Thus, it is not surprising that resveratrol is present in many food supplements. Resveratrol can either be extracted from plants (e.g., Japanese knotweed and grapes) or their products (e.g., wine) or obtained with biochemical and genetic engineering [11].

The aim of this study was to examine resveratrol food supplements purchased on the Slovenian market in the spring 2022 from two points of view: first, the compliance of declared resveratrol contents with the contents determined using high performance thin-layer chromatography (HPTLC), and second, the overall labeling compliance with EU regulatory requirements for mandatory and voluntary food information [3], nutrition and health claims on food [5], food supplements [1] and novel foods [4].

## 2. Materials and Methods

### 2.1. Chemicals

All chemicals were at least of analytical grade. Ethyl acetate, *n*-hexane, formic acid (98–100%), acetic acid (glacial, 100%) sulfuric acid (95–97%) and *p*-methoxybenzaldehyde (anisaldehyde) were from Merck (Darmstadt, Germany). Methanol (HPLC grade) was from J.T. Baker (Deventer, the Netherlands). Standard *trans*-resveratrol (99%) was purchased from Sigma-Aldrich (St. Louis, MO, USA).

### 2.2. Food Supplements with Resveratrol

**Figure 1:**
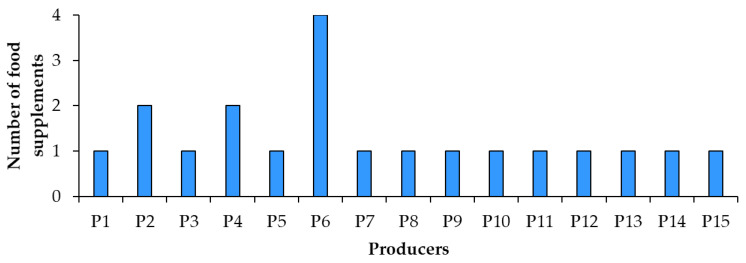
Number of food supplement products and producers.

**Figure 2:**
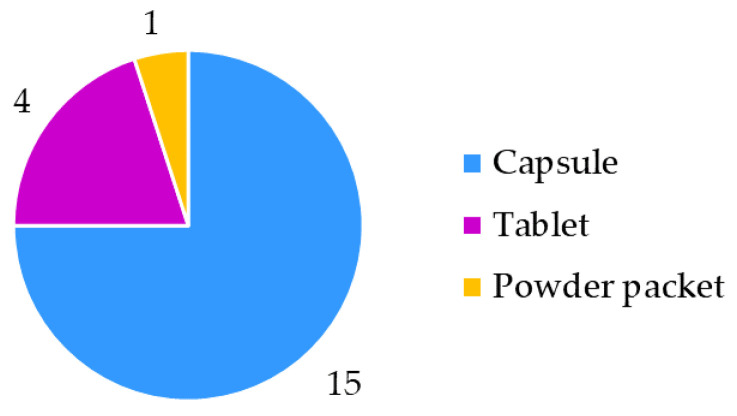
Forms of food supplement products.

In the spring of 2022, 20 resveratrol food supplement products were purchased in pharmacies and specialized stores in Slovenia. The food supplements were manufactured by 15 producers from 10 countries (4 samples from 1 producer, 2 samples from another 2 producers and only 1 sample from each of the remaining 12 producers) (Figure 1). Food supplements were in three forms (Figure 2): powder packet (sample 14), tablets (samples 1, 5, 6, 11) and capsules (the remaining 15 samples: 2, 3, 4, 7, 8, 9, 10, 12, 13, 15, 16, 17, 18, 19, 20). In addition to resveratrol, the food supplements analyzed in this study contained several other ingredients, such as plant parts (leaves, sprouts, rhizomes or roots, and fruits), plant extracts, carotenoids, amino acids, vitamins, minerals, etc.

### 2.3. Preparation of Standard Solutions

A standard stock solution of *trans*-resveratrol (1 mg/mL) was prepared in methanol. Working standard solutions of *trans*-resveratrol (0.1 mg/mL, 0.02 mg/mL, 0.01 mg/mL) were prepared by diluting a standard stock solution with methanol. All standard solutions were stored in amber glass storage vials at −80 °C.

### 2.4. Preparation of Sample Test Solutions (STSs) of Food Supplements

Sample test solutions (STSs) of food supplements were prepared using five units of each food supplement, where one unit was represented by one tablet or one capsule or one powder packet. The tablets were ground into powder in a grinder (mortar and pestle). The capsules were opened and the contents (powder or paste) were emptied. Each powdered or paste sample was homogenized by mixing. Afterwards each homogenized sample was applied for the extraction performed in three replicates. Homogenized powders or pastes of food supplement material were dispersed in methanol. The suspensions obtained were vortexed (5 s at 2800 rpm; IKA Vortex 1, IKA, Staufen, Germany) followed by ultrasound assisted extraction (15 min at 50 Hz; ultrasonic bath Sonis 3 GT, Iskra Pio d.o.o., Šentjernej, Slovenia). The supernatants obtained after 5 min centrifugation at 4200 rpm (Centric 322 A, Tehtnica, Železniki, Slovenia) were filtered through a 0.45 µm polyvinylidene fluoride (PVDF) membrane filter (Millipore, Billerica, MA, USA) into amber glass storage vials, which were stored at −20 °C. These undiluted STSs were then analyzed by HPTLC. Based on the differences in the declared contents of resveratrol in the food supplement samples STSs (triplicates) were prepared with the following concentrations: 0.2 mg/mL (samples: 1, 2, 4, 7, 8, 10, 12, 15, 19, 20), 1 mg/mL (samples: 3, 13, 17, 18) and 10 mg/mL (samples: 5, 6, 9, 11, 14, 16).

### 2.5. HPTLC Analyses

HPTLC analyses were performed on 20 cm × 10 cm glass backed HPTLC silica gel plates (Merck, Art. No. 1.05641). Standard solutions and STSs of food supplements were applied on the plates by means of an automatic TLC Sampler 4 (Camag, Muttenz, Switzerland). Applications were performed as 8 mm bands, 8 mm from the bottom of the plate and 15 mm from the left edge. For quantitative analysis the solution of *trans*-resveratrol standard (1, 2, 4, 8, 10 and 15 µL; 0.02 mg/mL) and three replicates of STSs of two food supplements were applied on each plate. Each of the three replicates of STSs was applied on the plate twice using data-pair technique (one application on the left and the other on the right half of the plate). Application volumes of STSs were as follows: 1 µL (samples 2, 4, 7, 8, 10, 11, 15, 17, 20), 2 µL (samples 1, 3, 9, 12, 13, 18, 19), 3 µL (sample 16), 5 µL (sample 6), 7 µL (sample 5) and 10 µL (sample 14). The plates were developed up to 9 cm in a saturated (15 min) twin-trough chamber (Camag) for 20 cm × 10 cm plates using 10 mL of the developing solvent *n*-hexane–ethyl acetate–formic acid (20:19:1, *v/v*) [12,13]. Development time was 25 min. After development and drying in a stream of warm air for 1 min, post-chromatographic derivatization was performed by dipping the plate for 2 s into anisaldehyde detection reagent by means of a Chromatogram immersion device III (Camag). Anisaldehyde detection reagent was prepared by mixing glacial acetic acid (20 mL) and methanol (170 mL). During cooling with cold water, 16 mL of sulfuric acid was added in a dropwise manner and subsequently anisaldehyde (1 mL) was added to the mixture [14] (pp. 195–196). Images of the plates were documented with a DigiStore 2 documentation system (Camag) at white light, 254 nm, 366 nm after development and after post-chromatographic derivatization. Densitometric scanning was performed by a slit-scanning densitometer TLC Scanner 3 (Camag) in the absorption/reflectance mode at 303 nm (before derivatization) or 500 nm (10 min after derivatization). The dimensions of the slit were: length 6 mm, width 0.3 mm; and the scanning speed 20 mm/s. All instruments were controlled by the winCATS software (Camag; Version 1.4.9.2001).

### 2.6. Validation of HPTLC Method

The HPTLC method for quantification of resveratrol (*cis*- and *trans*-resveratrol in one chromatographic zone) in food supplements was validated. Validation parameters included: system precision, limit of detection (LOD), limit of quantification (LOQ), linearity, accuracy (intraday precision and recovery) and were performed by the same person and laboratory equipment. Experimentally determined LOD was 10 ng, while LOQ was 20 ng. System precision was tested with nine applications of *trans*-resveratrol standard at 20 ng (LOQ) and at 80 ng (near the amount of resveratrol in applied test solutions of food supplements). Relative standard deviation (RSD) of resveratrol amount at 20 ng was 6.9%, while RSD at 80 ng was 4.4%, which met the established criteria (RSD < 10% at LOQ and RSD < 5% at the amount of resveratrol in the applied food supplement test solution). The regression coefficient for three polynomial calibration curves (20 ng, 40 ng, 80 ng, 160 ng, 200 ng and 300 ng) of resveratrol standard was 0.999, which satisfied the established criterion. Samples 12 (conc. 0.2 mg/mL), 18 (conc. 1 mg/mL) and 5 (conc. 10 mg/mL) were selected for testing the accuracy of the method. Six food supplement test solutions in each concentration were prepared simultaneously for intraday precision of the method. Three food supplement test solutions with addition of *trans*-resveratrol standard for each concentration were simultaneously prepared. Analysis for each concentration was performed on one HPTLC plate. Each solution with the addition of resveratrol standard was applied twice with six food supplement test solutions for intraday precision and resveratrol standard solution for calibration curve. RSD (*n* = 6) for intraday precision of food supplements test solutions prepared in 0.2 mg/mL was 5.49%, for food supplement test solutions prepared in 1 mg/mL was 4.58% and for food supplement test solutions prepared in 5 mg/mL was 4.60%. The obtained results met the established criteria (RSD < 10%). Recovery for sample 5 was 99.7%, for sample 18 97.1%, and for sample 12 102.3%. All values for recovery of all samples met the established criteria, which was 75–120%.

### 2.7. Label Regulatory Compliance

The EU regulatory compliance of the 20 food supplement products was checked for the following aspects: food labeling [3], health and nutrition claims [5], food supplements [1] and novel foods [4]. A checklist (Table 1) was created to see if all required information was provided on the labels and if the stated information was compliant with the requirements (units, fonts, necessary warnings and statements, etc.). Special attention was paid to any possible misleading practices that could have a negative effect on consumers.

## 3. Results and Discussion

### 3.1. HPTLC Quantification of Resveratrol and Assessment of Compliance of Declared and Determined Resveratrol Contents

**Figure 3:**
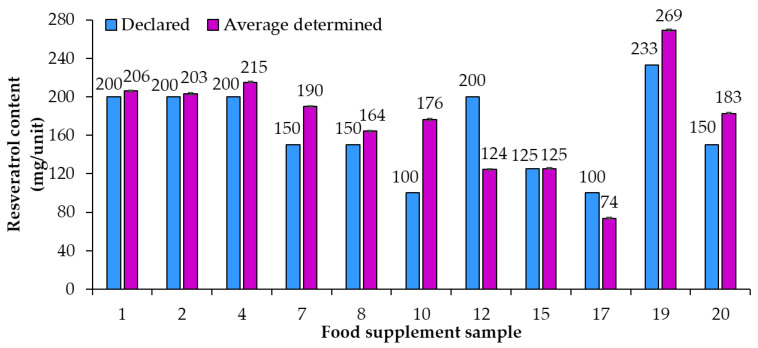
Comparison of declared contents vs. the average (*n* = 3) determined resveratrol contents in food supplements with contents of resveratrol higher than 70 mg/unit.

**Figure 4:**
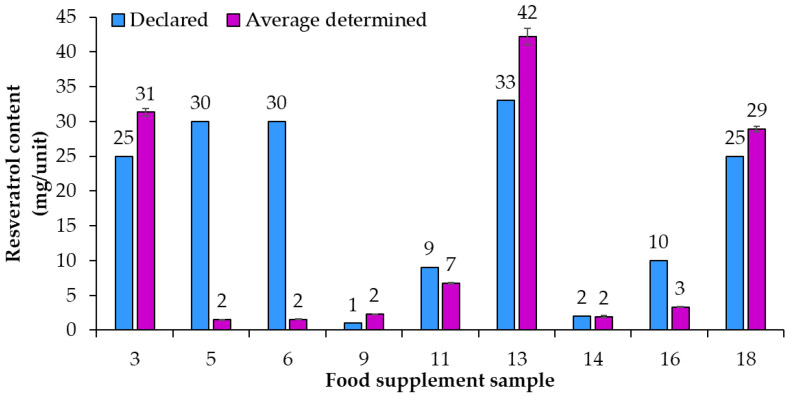
Comparison of declared contents vs. the average (*n* = 3) determined resveratrol contents in food supplements with contents of resveratrol lower than 50 mg/unit.

An overview of the 20 food supplement products included in this study showed that only seven of them (samples 1, 2, 7, 8, 11, 17, 18) were labeled to contain *trans*-resveratrol. For the remaining 13 samples it is, therefore, unclear if they contain a mixture of both isomers (*cis*- and *trans*-resveratrol, although only *trans*-resveratrol is approved as a novel food) or if the manufacturers chose to not write “*trans*-” and for a reason unknown declared what should be the *trans*-resveratrol content as resveratrol content. Therefore, it was decided to analyze the content of total resveratrol (both *cis*- and *trans*-resveratrol) within the scope of this study. HPTLC quantification of resveratrol was performed for 20 food supplement products claiming to contain resveratrol. Based on the results of the HPTLC analyses (Table 2) compliance of the declared resveratrol content with the average (*n* = 3) determined resveratrol content in food supplements was evaluated. For some samples the contents of resveratrol were higher than 70 mg/unit (Figure 3) and for others they were lower than 50 mg/unit (Figure 4). Various deviations of the average (*n* = 3) determined resveratrol contents from the declared contents (Figure 3 and Figure 4) were observed. The declared resveratrol contents ranged from 1 mg to 233 mg per unit (capsule, tablet, powder packet), while the average resveratrol contents determined ranged from 2 to 269 mg per unit (Figure 3 and Figure 4). In five food supplements (samples 1, 2, 4, 12, 19), representing 25% of the analyzed food supplements, the declared content was higher than 150 mg/unit, which the Commission Implementing Regulation (EU) 2017/2470 [4] sets as the maximum level (150 mg/day) for *trans*-resveratrol in food supplements. In eight food supplements (samples 1, 2, 4, 7, 8, 10, 19, 20), representing 40% of the analyzed food supplements, the average (*n* = 3) determined resveratrol contents were higher than 150 mg/unit (Table 2 and Figure 3). Of the 20 food supplements analyzed only one food supplement (sample 15) was found to have the same determined and declared resveratrol contents (Figure 3 and Table 2). In other words, in 95% of the analyzed food supplements the determined resveratrol content was either higher or lower than declared (Figure 3 and Figure 4). In twelve food supplements (samples 1, 2, 3, 4, 7, 8, 9, 10, 13, 18, 19, 20), representing 60% of the analyzed food supplements, the determined resveratrol contents were higher than declared (Figure 3 and Figure 4). In seven food supplements (samples 5, 6, 11, 12, 14, 16, 17), representing 35% of the analyzed food supplements, the determined resveratrol contents were lower than declared (Figure 3 and Figure 4).

**Table 2:** Average resveratrol content (mg/unit ± SD) determined in 20 food supplements.

Sample	Average Determined Resveratrol Content (mg/unit ± SD) ^1^	RSD (%) ^1^
1	205.92 ± 6.36	3.09
2	203.10 ± 1.56	0.77
3	31.35 ± 0.52	1.67
4	215.00 ± 0.89	0.41
5	1.52 ± 0.03	2.23
6	1.54 ± 0.05	3.15
7	189.85 ± 4.63	2.44
8	164.28 ± 1.92	1.17
9	2.34 ± 0.01	0.34
10	176.29 ± 4.37	2.48
11	6.78 ± 0.09	1.28
12	124.27 ± 3.28	2.64
13	42.21 ± 1.22	2.88
14	1.94 ± 0.13	6.49
15	125.41 ± 0.63	0.50
16	3.33 ± 0.07	2.01
17	73.76 ± 2.84	3.85
18	28.92 ± 0.37	1.28
19	269.14 ± 4.70	1.75
20	182.80 ± 3.48	1.90

^1^ *n* = 3.

**Figure 5:**
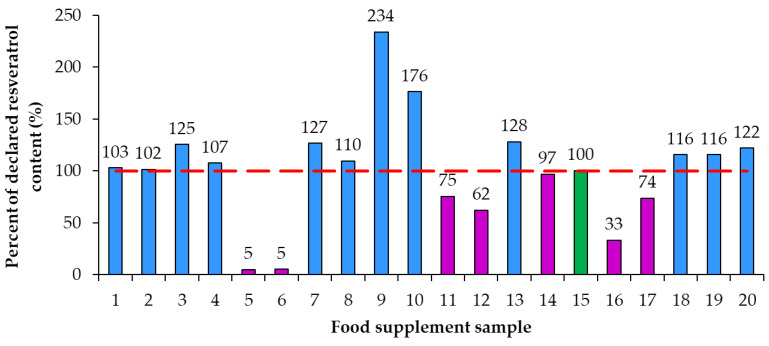
Percent of declared resveratrol content in food supplements.

**Figure 6:**
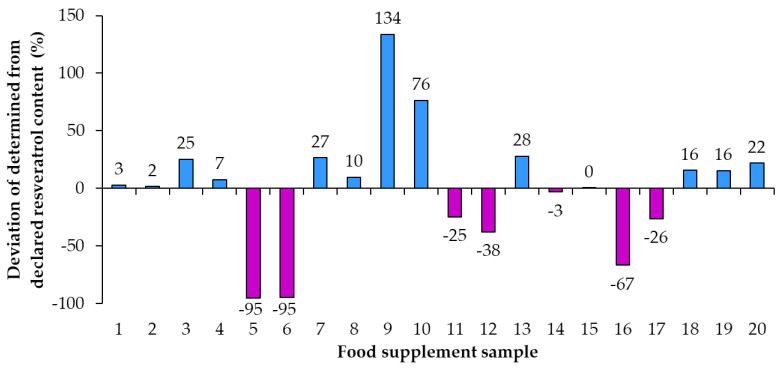
Deviation of determined content from declared content of resveratrol in food supplements.

Using the declared resveratrol contents and the contents of resveratrol determined by quantitative analyses, the percent of declared resveratrol contents in food supplements (Figure 5) and the deviation of determined content from declared content (Figure 6) were calculated. Only in sample 15 was the percent of declared resveratrol content 100% (Figure 5). In the remaining 95% of resveratrol food supplements the determined contents of resveratrol were different from the declared contents and consequently percent of the declared resveratrol content were higher or lower than 100% with differences ranging from 5% to 234% (Figure 5). In five food supplements (samples 1, 2, 4, 8, 14), representing 25% of the food supplements analyzed, the deviation of determined content from declared content (Figure 6) was 10% or less than 10% (percent of declared resveratrol contents ranged between 97% and 110%, Figure 5). In 14 food supplements (samples 3, 5, 6, 7, 9, 10, 11, 12, 13, 16, 17, 18, 19, 20), representing 70% of the food supplements analyzed, the deviation of determined content from declared content (Figure 6) was higher than 10% (more than 10% higher or 10% lower than declared). In eight of those 14 food supplements (samples 3, 7, 9, 10, 13, 18, 19, 20), representing 40% of the food supplements analyzed, the percent of declared resveratrol contents ranged between 116% and 234% (Figure 5). In the other six of those 14 food supplements (samples 5, 6, 11, 12, 16, 17), representing 30% of the food supplements analyzed, the percent of the declared resveratrol contents ranged between 5% and 75% (Figure 5). In four food supplements (samples 5, 6, 12, 16), representing 20% of the food supplements analyzed, deviations of determined contents from declared contents showed that determined resveratrol contents were more than 30% lower than declared contents (Figure 6). In two samples (samples 5 and 6), representing 10% of the food supplements analyzed, the deviation of determined content from declared was −95% (Figure 6) as the products contained only 5% of the declared resveratrol content (Figure 5).

As evident from the calculated deviations of determined contents from declared contents presented in Figure 5, the determined resveratrol contents for 30% of the food supplements were more than 30% higher/lower than the declared contents. Samples 9 and 10, representing 10% of the food supplements analyzed, had the deviation of the determined from the declared resveratrol content higher than 130% and 70%, respectively (Figure 5). Such deviation can have different consequences for consumers (Figure 5). The declared content of sample 9 was 1 mg, but the determined content was 2.34 mg (Figure 4, Table 2), which means that, although the content declaration was not providing accurate information to the consumer, there is a small likelihood that this could result in adverse health effects for the consumer. The declared content of sample 10 was 100 mg, but the determined content was actually 176 mg (Figure 3, Table 2), which is even higher than the permitted daily intake of 150 mg of *trans*-resveratrol. The determined content in sample 10 poses an even higher risk for the consumer because taking too much resveratrol can lead to health issues (e.g., digestion problems). Therefore, from a food safety perspective, the differences between declared and determined content of resveratrol are not just misinforming consumers, as high concentrations can also result in negative effects on consumer health.

In two other studies of resveratrol in food supplements the authors reported similar deviations of the determined and declared contents [15,16] and also contents (declared and/or determined) higher than 150 mg per unit. In the first study authors analyzed *trans*-resveratrol in 28 food supplements and reported that in 17 food supplements, representing more than 60% of food supplements analyzed, the determined contents were lower than declared contents [15]. Additionally, in the remaining 11 food supplements, representing more than 39% of food supplements analyzed, the determined contents were higher than declared contents [15]. Differences between the declared contents and determined contents were ranging from 24% to 262% [15]. For 17 food supplements the deviations of determined contents from declared contents were up to ±10% [15]. In six food supplement products, representing more than 20% of the food supplements analyzed, the declared and determined contents were higher than 150 mg/unit [15]. In the second study the authors analyzed resveratrol in nine food supplements and found that the determined resveratrol contents were lower in eight samples and higher than the declared contents in one sample [16]. The declared resveratrol contents ranged between 10 and 300 mg per unit, while the determined contents ranged between 3.4 and 147.3 mg per unit. The determined resveratrol contents ranged between 22.8% and 104.7% of the declared contents [16]. Three food supplements, representing 33.3% of the food supplements analyzed, contained less than 60% of the declared contents [16]. The deviation from the declared resveratrol content in one food supplement was 77%. In two food supplements the declared resveratrol contents were higher than 150 mg per unit (250 mg and 300 mg), however, the determined contents were lower than 150 mg per unit (144.9 mg and 147.3 mg) [16]. Surprisingly, a recently published list of products containing 20 to 1400 mg of resveratrol per serving included more than 45% of the products with the declared resveratrol contents higher than 150 mg/unit [17].

Issues of noncompliance of the declared and the determined contents were recently also reported for food supplements claiming to contain folic acid [18], vitamin A [19], vitamin C [19], vitamin E [19] and magnesium [20]. Analyses of 30 folic acid food supplements available on the Polish market revealed that in 29 products, representing 96.6% of food supplements analyzed, the determined contents were lower than the declared [18]. Seventeen food supplements, representing 56.6% of food supplements analyzed, contained less than 80% of the declared folic acid content. Nine food supplements, representing 30% of food supplements analyzed, contained less than 50% of the declared folic acid content [18]. Four food supplements, representing 13.3% of food supplements analyzed, contained less than 3% of the declared content of folic acid [18]. In contrast the analysis of folic acid content in the food supplements sold in Spain [21] showed that the determined content was within a tolerated range with regard to the declared values, which were in accordance with EU regulation requirements. Noncompliances of the declared and determined contents were also found in a study of vitamins A, C and E in 57 food supplements available on the Brazilian market [19]. The determined contents for vitamins A and E were lower than the declared contents in 71% and 50% of food supplements, respectively [19]. In another study of 116 food supplements containing magnesium the authors reported noncompliance of the declared and determined contents for 58.7% of the products analyzed [20]. In only two samples, representing 1.7% of the food supplements analyzed, the declared and determined contents were identical [20].

The results of this study of resveratrol in food supplements and the results of studies performed by other authors for resveratrol or other declared ingredients indicate the need for improved quality control by food supplements producers (including stability studies of the ingredients) as well as more frequent control by regulatory authorities to ensure the reliable declarations for consumers.

### 3.2. Label Regulatory Compliance

**Figure 7:**
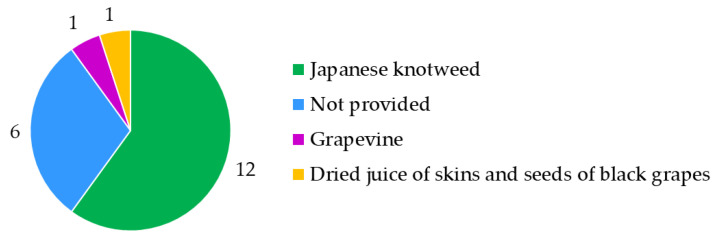
Resveratrol origin declared on food supplements.

Label regulatory compliance of 20 food supplement products sold on the Slovenian market and claiming to contain resveratrol or *trans*-resveratrol from different sources was evaluated. The origin of resveratrol (Figure 7) was not provided on six products. Most food supplements (12 products) listed Japanese knotweed and some products even specified the plant’s rhizomes as the source of resveratrol. The other food supplements listed grapevine (1 product) or dried juice of skins and pips of black grapes (1 product) as the source of resveratrol. The analyzed food supplements were produced by 15 producers (Figure 1), three of which produced more than one product which replicated compliance and noncompliance (errors) across their food labels.

#### 3.2.1. Mandatory Food Information

**Figure 8:**
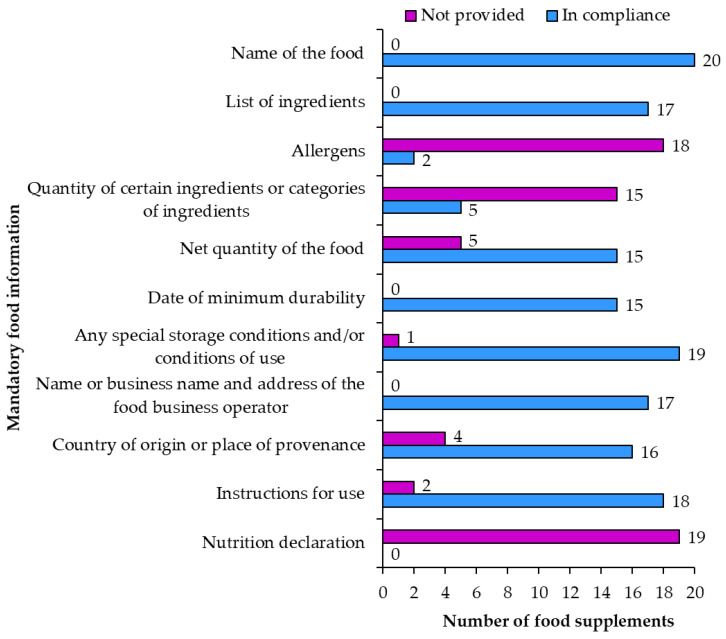
Compliance of mandatory food information on food supplement labels.

Not all of the 12 mandatory requirements [3] for food information are mandatory for food supplements. The noncompliances noted in the overview of mandatory food information (Figure 8) varied from grammatical mistakes to mistakes that could mislead or negatively influence the consumer.

**Name** of the food was written correctly on all 20 food supplement products.

**List of ingredients** was accurately labeled on 17 food supplement products (1, 2, 5, 6, 7, 8, 9, 10, 11, 12, 13, 14, 16, 17, 18, 19, 20). For most samples the list of ingredients began with a title that included the word “ingredients” followed by a list of ingredients. Lists of ingredients on two samples did not have the word “ingredients” in the title. One sample did not include a list of ingredients, but only listed ingredients “per capsule”. Ingredients should be listed in descending order of their mass. Due to lack of information, it was not possible to check if the order was correct (descending) for all the ingredients. For some ingredients (such as vitamins, minerals and resveratrol) the quantities were provided on the labels and it was discovered that these ingredients were not included on the ingredients list according to the descending mass. Labeling of nano materials was compliant with regulatory requirements with the word “(nano)” after the ingredient in both samples (3 and 4). Some lists of ingredients were written in a way that the title “Ingredients” was followed by words “one capsule contains” or “three capsules contain” and then the list of ingredients. There was only one sample where the list of ingredients was unclear and confusing as the list was repeated two times: first the list titled “Ingredients in one capsule” (which included “Japanese knotweed rhizomes extract that contains resveratrol”) and second list titled “Ingredients” (which included the whole list of ingredients including “knotweed that contains resveratrol”). Labeling of additives showed a trend of avoiding writing additives with their E-numbers. All additives on all food supplements were correctly labeled with their functional classes and names. Perhaps this trend reflects the consumers dislike of E-numbers that can be connected to bad reputations of some additives. However, writing additives with their names instead of using the E-numbers does not mean that additives are not present in food supplement products. One food supplement even emphasized the information “additive free”. On multiple food supplements the adjective “natural” was used when describing the ingredients (e.g., “natural *trans*-resveratrol (from a knotweed species, *Polygonum cuspidatum*)”, “natural caramel color”, “natural *trans*-resveratrol from Japanese knotweed”, “capsule: tapioca gelatin, naturally fermented in pullulan”, “natural *trans*-resveratrol”, “natural beta-carotene/mixed carotenoids”, etc.). The EU legislation is not very clear about the use of the adjective “natural” on food labels, as the term “natural” is only mentioned in cases of flavoring substances (Regulation (EC) No 1334/2008 [22]) and the nutrition claim “natural” [5]. Flavoring substances can be labeled as “natural” if they fulfill the requirements and do not mislead consumers. The nutrition claim “natural” can only be used for natural properties of foods. The current regulation of using the term “natural” on food labels should be improved because there is too much room for consumer confusion and misunderstanding.

**Allergens** were correctly labeled (e.g., “lecithin (**soy**)” or “**soy** lecithin”) on two products (samples 5, 14) and not provided on 17 products. An ingredient (“**fish** oil”) was not written correctly, as allergens should be written in a different font (e.g., “fish oil”). There was also some confusion with correctly written ingredient “phytosterols (from **soy**)”, which was followed by “Information about allergens: contains soy (emphasized above)”. The last statement was not needed, and even if considered as an attempt at voluntary food information, it only confuses the consumer.

**Quantity of certain ingredients or categories of ingredients** was correct on five products (1, 2, 9, 13, 20) as the quantity of resveratrol was provided in percent in the list of ingredients. The rest of the products did not include this information on their labels, which is alright because this information is only required in case its absence could result in consumers being misled. In most cases, the quantities of ingredients such as vitamins and minerals (when emphasized with a word or image next to the products name) were provided only among the ingredients or in a table of vitamin and mineral content which is compliant with the food supplement regulation [1] and is not regulated as “quantity of ingredients” by food labeling regulation [3].

**Net quantity of food** is mandatory on all food labels. Net quantity of the food was accurately labeled with appropriate units on 15 products (samples 1, 2, 3, 4, 5, 6, 7, 9, 10, 11, 12, 13, 14, 19, 20) and not included on five products.

**Date of minimum durability** is mandatory on all food labels, but was properly written on only 15 products (samples 1, 3, 4, 6, 7, 9, 10, 11, 13, 14, 15, 16, 17, 19, 20). Other products had different errors in the writing of the date on the label. The date should be provided with the words “Best before …” when the date includes an indication of the day or “Best before end …” in all other cases followed by the date or the location of the date on the food packaging. An example of an unclear date labeling was “Expiry date (use by Do not consume the enclosed preservative! Leave it in the bottle. Expiry date (Best before end:) and serial number are printed on the bottom of the bottle.” On another product the “Best before end” was not written and only the date was printed. There was also a product where the date was written without punctuation (“1023”), which is confusing for the consumers. Even more unclear was the writing on the sample in which the lot or batch number and date were written together with no space or punctuation. Another product had a correctly written date, but the location of the date was not as specified. Yet another product had a correctly written date, but the location of the date was not provided, although required for products where “Best before” is not followed by the date. Although lot numbers (batches of sales units manufactured under almost the same conditions) are not a part of mandatory food information regulation, marketing of foodstuffs requires lot numbers on labels (Directive 2011/91/EU [23]). Lot numbers were provided on 18 products (1, 2, 3, 4, 5, 6, 7, 9, 10, 11, 12, 13, 14, 15, 16, 17, 19, 20).

**Special storage conditions and/or conditions of use** were provided in accordance with requirements on 19 (samples 2, 3, 4, 5, 6, 7, 8, 9, 10, 11, 12, 13, 14, 15, 16, 17, 18, 19, 20) of 20 food supplements. One sample did not provide this information. Examples of special storage conditions were “store at a temperature up to 25 °C protected from moisture and light”, “store in a dry place at room temperature” and “store well closed”. Examples of conditions of use included “only for adults”, “in case of gastrointestinal unease stop using the product”, “not suitable for: pregnant women, breastfeeding mothers and children younger than 12” and “in case of taking immunosuppressants or other prescribed medication consult your doctor about taking this product”.

**Name or business name and address of the food business operator** were correctly labeled on 17 food supplements (samples 1, 2, 5, 6, 7, 8, 9, 10, 11, 12, 13, 14, 15, 17, 18, 19, 20). The irregularities of writing the business name and address included: not writing the complete address, not writing the address at all and writing the town twice instead of the full address.

**Country of origin or place of provenance** is only strictly mandatory in cases in which the lack of this information could result in misleading consumers. Country of origin was correctly written on 16 (samples 1, 2, 3, 4, 7, 8, 9, 10, 11, 12, 13, 14, 17, 18, 19, 20) and not included on four food supplement products. In most cases this information was provided with the words “Origin”, “Produced in” or “Manufactured in”. Most products were produced in the UK, France and USA.

**Instructions for use** were properly provided on 18 food supplements (samples 1, 3, 4, 5, 6, 7, 8, 9, 10, 11, 12, 14, 15, 16, 17, 18, 19, 20) and not included on two food supplements. For sample 14, the product in the form of a powder packet, the instructions were: “put the contents of the packet in a glass or bottle with water (0.2–0.5 L)”. Food supplements in the form of capsules or tablets included instructions to take them with liquid (water or juice) and to take them before/during/after a meal in the morning or evening.

**Nutrition declaration** was not included on 19 of the 20 food supplements. The label of one sample included a nutrition declaration, which was mostly correct, but the energy value units were incorrect.

#### 3.2.2. Voluntary Food Information

**Figure 9:**
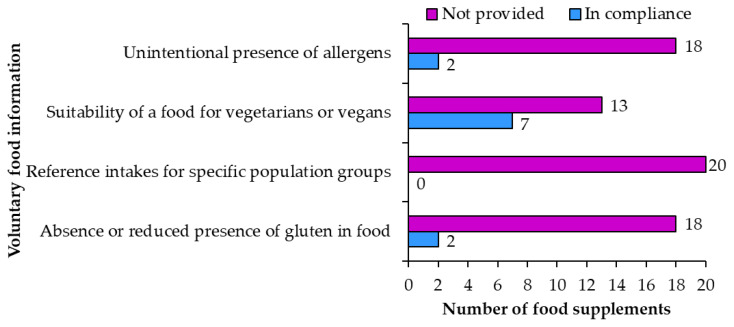
Voluntary food information on food supplement labels.

**Voluntary food information** is also regulated [3]. The labels of examined food supplements only included voluntary food information in nine (samples 2, 7, 8, 10, 11, 14, 16, 17, 18) of 20 food supplements (Figure 9).

An **unintentional presence of allergens** was appropriately labeled on two food supplements (samples 2, 11) and not included on the remaining 18. For example, “May contain traces of sulfites.” It was noticed that in some cases unintentional presence of allergens was not labeled properly. The irregularities ranged from writing in bold (a different font should only be used for the mandatory labeling of allergens presence) to incorrect use of allergen terminology (for example “nuts” instead of “walnuts”).

**Suitability of a food for vegetarians or vegans** was correctly written on seven products (samples 2, 7, 8, 14, 16, 17, 18) and not provided on 13. One product had the statement about vegetarian/vegan suitability written in English on the original label but was not translated and included on the Slovene label. This is not an irregularity, but it can be confusing for consumers who know both languages and read all the labels.

**Reference intakes for specific population groups** were not provided on any of the 20 food supplement products.

**Absence or reduced presence of gluten** in food was correctly labeled on two products (samples 10, 14) as “gluten free”. The remaining 18 products did not have this information. The use of “gluten free” is allowed on foods produced in ways that they do not contain gluten. It is also allowed on foods from ingredients that naturally do not contain gluten. This information should not mislead the consumers that the food product has special properties if similar foods also have the same properties [6].

#### 3.2.3. Nutrition and Health Claims

**Figure 10:**
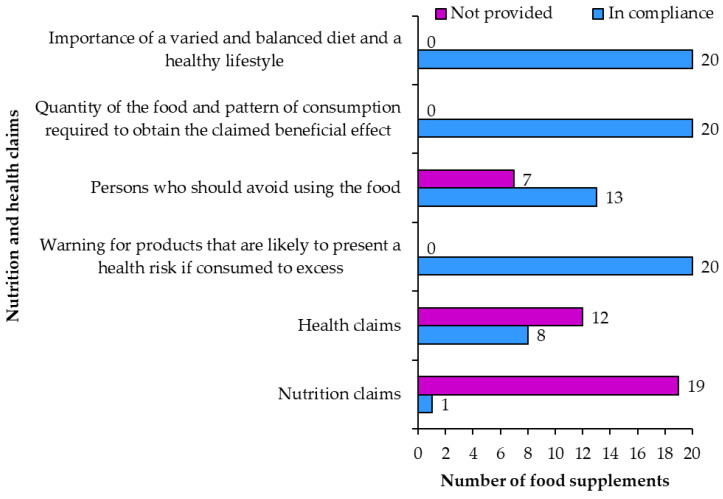
Food supplements with health and nutrition claims.

**The use of nutrition and health claims** (Figure 10) is voluntary [5]. Nutrition claims were only included on one product (sample 10: “salt-free”). Salt content of the product was not included on the label of sample 10. Other resveratrol food supplements also did not contain salt—making “salt-free” a shared property of similar products. The fact that sample 10 is labeled salt-free does not make the products salt content different from similar products. The use of the “salt-free” nutrition claim is permitted if the product does not contain more than 0.005 g of sodium (or the equivalent amount for salt) per 100 g. Therefore, the use of the “salt-free” nutrition claim is misleading. Two more samples had the claim “sugar free” on the original English label, but the Slovenian label did not have this claim.

**Health claims** were written on eight products (samples 3, 4, 5, 6, 9, 13, 14, 19). Together these eight products contained 74 health claims. Health claims connected the vitamins and minerals in food supplements with the following health effects: protecting cells from oxidative stress, reducing fatigue and exhaustion, functioning of the muscles, heart, nervous and immune system, maintaining vision and healthy bones, releasing energy during metabolism (Table 3).

On some food supplement labels, health claims were written using the same words as on the list of the approved claims [24]. On other products the claims were slightly paraphrased in a way that did not change the meaning of the health claim or the consumer’s understanding of the claim. Therefore, it can be concluded that the use of health claims was in compliance with regulatory requirements for all eight samples.

When using nutritional and/or health claims the labels must also include the statements: about the importance of a varied and balanced diet, the amount of food and the required method of consumption and warning about the danger of excessive consumption. The warning for products that are likely to present a health risk if consumed to excess was accurately included on all 20 food supplement labels. A warning for persons who should avoid the food was correctly written on 13 food supplements (samples 2, 3, 4, 5, 6, 8, 10, 12, 13, 16, 17, 18, 19) and not included on the remaining seven food supplements. The labels of all products included the quantity of the food and pattern of consumption required to obtain the benefits claimed as well as the statement regarding the importance of a varied and balanced diet and a healthy lifestyle.

Health claims were also evaluated for regulatory compliance in a study of folic acid food supplements sold in Spain [21]. The results revealed that the food supplements sold in supermarkets fulfilled the requirements for folic acid health claims, while over 14% of food supplements sold online did not fulfill the requirements for folic acid health claims as they contained nonauthorized health claims.

**Table 3:** Health claims on food supplement labels grouped according to the related nutrients.

Nutrients (Number of Products Using the Health Claim)		Health Claims (Summarized from Commission Regulation (EU) No 432/2012 [24]
Copper (2), zinc (2), manganese (1), selenium (3), vitamin C (3), vitamin E (1)		[Nutrient] contributes to the “protection of cells from oxidative stress.”
Magnesium (3), niacin (3), pantothenic acid (3), vitamin B2/riboflavin (3), vitamin B6 (3), vitamin B12 (3), vitamin C (2)		[Nutrient] contributes to the “reduction of tiredness and fatigue.”
Calcium (1), potassium (1), magnesium (2), vitamin D (2)		Nutrient] contributes to “normal muscle function.”
Thiamine (1)		[Nutrient] contributes to the “normal function of the heart.”
Copper (1), zinc (2), folate (2), selenium (1), vitamin B6 (2), vitamin B12 (2), vitamin C (2), vitamin D (3)		[Nutrient] contributes to the “function of the immune system.”
Copper (1), biotin (1), iodine (1), potassium (1), magnesium (2), niacin (1), vitamin B1/thiamine (1), vitamin B2/riboflavin (1), vitamin B6 (1), vitamin B12 (1), vitamin C (1)		[Nutrient] contributes to “normal functioning of the nervous system.”
Zinc (1)		[Nutrient] contributes to the “maintenance of normal vision.”
Calcium (1), vitamin D (1)		[Nutrient] contributes to the “maintenance of normal bones.”
Magnesium (1), niacin (1), vitamin B2/riboflavin (1), vitamin B6 (1), vitamin B12 (1)		[Nutrient] contributes to “normal energy-yielding metabolism.”

#### 3.2.4. Food Supplements and Novel Foods

**Figure 11:**
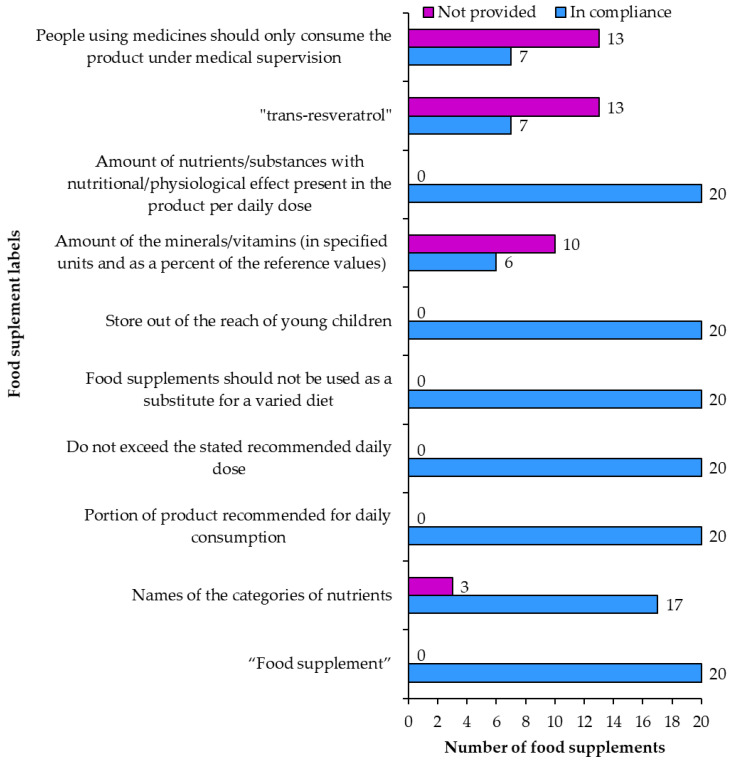
Food supplement information and novel food information on food supplement labels.

According to food supplements regulation [1] products are required to be labeled as “food supplements”. All 20 of the examined products fulfilled this requirement (Figure 11). The labels of all 20 products included the information that the products contained resveratrol. Seventeen of these products (samples 2, 3, 4, 5, 6, 8, 9, 11, 12, 13, 14, 15, 16, 17, 18, 19, 20) also included the names of other categories of nutrients while labels of the remaining three products did not include this information (Figure 11). All 20 products were labeled with the following **required warnings** (Figure 11): (1) not to exceed the stated recommended daily dose, (2) that food supplements should not be used as a substitute for a varied diet, and (3) store out of reach of young children. All 20 products also included the information on the portion of product recommended for daily consumption. Five products had unusual labels regarding the recommended daily dose “The recommended daily amount or dose should not be exceeded, unless your doctor has instructed you to do so”.

The **amounts of minerals/vitamins** in specified units and as a percentage of the reference values were correctly provided on six (samples 3, 4, 9, 13, 14, 17) and not provided on 10 food supplements (Figure 11). For example, the amount of copper was labeled in mg instead of µg, which should be used according to the regulation [3]. Some samples used the wrong order of ingredients on the list of ingredients not following the descending order of mass.

The **amounts of nutrients/substances** (mainly resveratrol) with nutritional/physiological effect, present in the product and per daily dose were written correctly on all 20 products (Figure 11).

**Figure 12:**
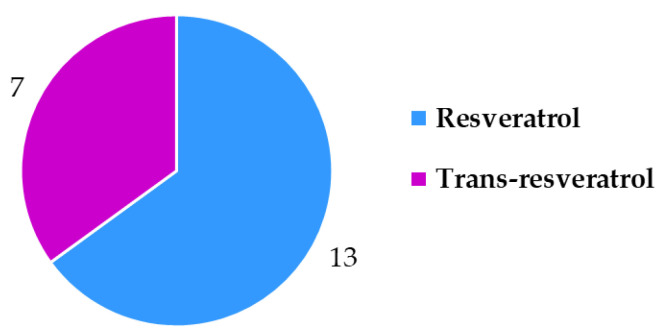
Resveratrol vs. *trans*-resveratrol declarations on food supplement labels.

The field of **novel food labeling** regulatory [4] compliance proved to be far more challenging than food supplement labeling (Figure 11). The regulation only requires two things for resveratrol food supplements: to be labeled as “*trans*-resveratrol” and to have a warning that people using medicines should only consume the product under medical supervision. Only seven products (samples 1, 2, 7, 8, 11, 17, 18) were labeled to contain “*trans*-resveratrol” and the others usually referred to this compound as only resveratrol (Figure 12). The medical warning about people on medication to take the supplements under a doctor’s supervision was only written on seven food supplements (samples 2, 6, 8, 12, 16, 17, 18). In terms of novel food labeling for resveratrol food supplements only four of the products were labeled correctly (samples 2, 8, 17, 18).

#### 3.2.5. Other Label Noncompliances

Other noncompliances with the regulation concerning food supplement labeling were also observed such as: typos, use of misleading images, nonauthorized health and nutrition claims as well as overuse of voluntary information.

Typos were a common type of error on food supplement labels. In most cases this included wrong punctuation (swapping periods and commas or incorrect placement of commas and semicolons). On one product words were written in the middle of the date of minimum durability, which made it impossible to read and understand the date. On another product the label included the wrongly spelled word “resveratrol” (it was written “rezervatrol” instead). Such examples may seem innocent, but they can have negative effects on consumers.

Misleading use of photos was observed on several labels. On one product a picture of fruit (apple, pear, lemon and orange slices stacked on top of each other) was placed on the largest side of the cardboard box and while the ingredients did not include these fruits, the manufacturer tried to solve this by writing “The product does not contain the fruit in the picture.” next to the photo. Despite this claim, the use of such graphics can mislead consumers. Another product clearly listed Japanese knotweed as the source of resveratrol, but the front of the packaging had an image of mint, which was not listed among the ingredients. This kind of image use could also mislead the consumers. On another product with resveratrol from Japanese knotweed, there was a picture of grapes, which were not present on the list of ingredients.

Another problematic practice was the use of nonauthorized health claims linking food supplements with resveratrol and in some cases also other ingredients with positive health effects. Examples of these claims included: “resveratrol contributes to a healthy cardiovascular system”, “natural antioxidant”, “preserving youth for smooth skin” and “lignans from flaxseed may support a healthy prostate in aging men”.

There were also differences between the information provided on Slovene and English language labels. For example, one product’s original label in English language included nonauthorized health claims, which were not translated and included on the Slovenian label. This is both good and bad, because the Slovenian label was in accordance with the regulation regarding health claims, but at the same time consumers knowing both languages could be confused due to the different label contents. There were several other cases in which there were differences between the original (mostly English) and translated (Slovenian) labels. These differences included statements claiming that resveratrol can help to support healthy cardiovascular functions, has cellular anti-aging properties, has the ability to promote a healthy response to biological stress, supports healthy aging, supports antioxidant health, etc. In some cases, these statements were followed by a disclaimer that the statements were not evaluated by the FDA and that the product is not intended to diagnose, treat, cure or prevent any disease.

Statements about not containing certain ingredients were often used. Regulation 1169/2006 [3] permits highlighting special properties of food products, except if similar food products also have the same properties. The use of “lactose free” was not done correctly as its use is only limited to products containing milk or milk products or ingredients. The use of “Lactose free” requires labeling of an actual content of lactose in g/100 g of product. Some food supplement labels claimed not to contain certain ingredients such as: fillers, binders or other excipients, gluten, wheat, lactose, added sugar, sugar, salt starch, soy, milk, lactose, yeast, titanium dioxide, artificial flavorings or colorings.

The labels of some food supplement products included too much voluntary information. Probably the purpose of that was to attract consumers’ attention and convince them to purchase the product. However, because these voluntary claims took a larger portion of the label, the legitimacy of using them is questionable as they can confuse the consumer and make it difficult for consumers to access the mandatory food information. Examples of those claims included: descriptions of holistic innovations, procedures for the plant ingredients preparations, comments about research results, etc.

The most important aspect and purpose of food labels in the EU is to provide consumers with understandable information. Clearly the evidence shows that there are still many improvements needed to achieve the goal of clear and comprehensive labeling of food supplements that fulfill all regulatory requirements.

## 4. Conclusions

Various deviations of the average determined contents of resveratrol from the declared contents were found. Only one of 20 food supplement products analyzed was found to have the same content as declared. Seven products were found to have a lower content of resveratrol and 12 products a higher content of resveratrol. The determined contents ranged from 5% to 234% of declared content. In 40% of the food supplement products analyzed the determined content even exceeded the maximum level (150 mg/day) for *trans*-resveratrol in food supplements set by EU regulation, which can have negative effects on the health of consumers of these food supplements. From a food safety point of view, the differences between declared and determined content of resveratrol in food supplements are concerning.

Resveratrol food supplement labels are very diverse in both style and the information they provide. The results of this label regulatory compliance overview showed that most labels contained the necessary information and that in most aspects labels followed the regulatory requirements for mandatory and voluntary food information, health and nutrition claims as well as food supplement and novel food labeling. However, labels also contained multiple errors (ranging from typos—even resveratrol—to deceptions) that could have different consequences for the consumers as some errors may confuse and misinform them. Particularly concerning were irregularities that were the result of negligence (such as writing the lot number together with the date of minimum durability) and the misleading use of images on the food supplement packaging (e.g., fruit or mint on products that do not contain fruit or mint as well as grapes on a product not made from grapes but Japanese knotweed).

Labels incorrectly provided information about the possible presence of allergenic substances, which could be present due to contamination, as allergenic substances were written in a different font or not represented correctly (e.g., lactose free). The use of too much voluntary information was also a problematic practice as this information took most of the space on the label of some products, which already used very small fonts, making the label information overwhelming and confusing for consumers. Voluntary claims about the absence of certain ingredients (e.g., lactose free, yeast free, salt free, sugar free) were quite common, but in most cases, they were not used appropriately as they referred to ingredients that were not present in any of the food supplement samples. Hence the absence of those ingredients was not a different property (e.g., lactose free or salt free) compared to other similar products. Labels also contained nonauthorized health claims connecting resveratrol and other ingredients with positive health effects (e.g., preserving youth—for smooth skin).

Labels were often translated containing both the original (printed) label and the translated label (on a sticker). In many cases the contents of the original and translated labels were different (e.g., not all information was translated from English to Slovene, which could be confusing for consumers understanding both languages, but in some cases these differences made Slovenian labels compliant with the regulation where English labels were not compliant).

Labels should be improved to reduce the number of truly unnecessary errors and to be fully compliant with the regulatory requirements as well as become more comprehensive for the consumers. Both responsible food business operators and regulatory or inspection bodies should dedicate their attention to improving food supplement labels. There is still a lot of room for improvements. Perhaps it would also be beneficial for regulations to specify some aspects of food labeling in more detail (e.g., use of voluntary claims in relation to scientific evidence or lack thereof and the use of the term “natural”). An increase of minimum font size should be considered by the regulatory body. Present minimum font size and the abundance of text on food supplement labels often result in labels that are extremely difficult to read due to the density of text and use of far too small font size. After all, everyone is a consumer and deserves to have labels with clear information about food supplement products because that helps consumers to choose the right product for them.

## Figures and Tables

**Table 1:** Regulatory compliance checklist for food supplement labels for the regulation topics of food labeling, health and nutrition claims, food supplements and novel foods.

Regulatory Compliance Checklist for Food Supplement Labels	
Regulation Topic	Information
Mandatory food information	Name of the food
	List of ingredients
	Allergens
	Quantity of certain ingredients or categories of ingredients
	Net quantity of the food
	Date of minimum durability
	Any special storage conditions and/or conditions of use
	Name or business name and address of the food business operator
	Country of origin or place of provenance
	Instructions for use
	Nutrition declaration *
Voluntary food information	Absence or reduced presence of gluten in food
	Reference intakes for specific population groups
	Suitability of a food for vegetarians or vegans
	Unintentional presence of allergens
Nutrition and health claims	Nutrition claims
	Health claims
	Warning for products that are likely to present a health risk if consumed to excess
	Persons who should avoid using the food
	Quantity of the food and pattern of consumption required to obtain the claimed beneficial effect
	Importance of a varied and balanced diet and a healthy lifestyle
	Nutrition declaration
Food supplements	“Food supplement”
	Names of the categories of nutrients
	Portion of product recommended for daily consumption
	Do not exceed the stated recommended daily dose
	Food supplements should not be used as a substitute for a varied diet
	Store out of the reach of young children
	Amount of the minerals/vitamins (in specified units and as a percentage of the reference values)
	Amount of nutrients/substances with nutritional/physiological effect present in the product & per daily dose
Novel foods	“*trans*-resveratrol”
	People using medicines should only consume the product under medical supervision

* Mandatory only for food supplements with nutrition and/or health claims.

## Data Availability

The data presented in this study are available on request from the corresponding author.
